# Supplementary material for: Differential diagnosis of a calcified cyst found in an 18th century female burial site at St. Nicholas Church cemetery (Libkovice, Czechia)
Source: PLoS One. 2021 Jul 2;16(7):e0254173. doi: 10.1371/journal.pone.0254173 (PMC8253445; doi:10.1371/journal.pone.0254173)
Supplement: Attachment — Submitted filename: Response to Reviewers.pdf [file pone.0254173.s001.pdf]

UNIWERSYTET PRZYRODNICZY WE WROCŁAWIU

INSTYTUT BIOLOGII ŚRODOWISKOWEJ
ZAKŁAD ANTROPOLOGII

Wrocław, 15.05.2021

Response to Reviewers

Dear Sir or Madam,

On behalf of the authors of the article *'Differential diagnosis of a calcified cyst found in an 18th century female burial site at St. Nicholas Church cemetery (Libkovice, Czechia)'* I would like to express our sincere thanks for your work on the formal evaluation and the substantive review of the article. We were pleased to receive the information that the content of the article met your approval, and we considered the minor revisions proposed by you as well-thought-out and highly valuable for the article.

At the outset, we are obliged to explain that due to the formal changes that the Department of Anthropology has undergone from the moment of submitting the article (March 2021), we are obliged to correct the affiliation of authors associated with Wrocław University of Environmental and Life Sciences. The current affiliation of these authors is: Wroclaw University of Environmental and Life Sciences, Institute of Environmental Biology, Division of Anthropology.

According to Academic Editor's comments:

1. *'Please ensure that your manuscript meets PLOS ONE's style requirements, including those for file naming. The PLOS ONE style templates can be found at https://journals.plos.org/plosone/s/file?id=wjVg/PLOSOne_formatting_sample_main_body.pdf and https://journals.plos.org/plosone/s/file?id=ba62/PLOSOne_formatting_sample_title_authors_affiliations.pdf'*

We carefully checked the PLOS ONE's style requirements and after our recent corrections we hope that our article meets all the journal's standards for publication right now.

2. *'In your manuscript, please provide additional information regarding the specimens used in your study. Ensure that you have reported specimen numbers and complete repository information, including museum name and geographic location.*

   If permits were required, please ensure that you have provided details for all permits that were obtained, including the full name of the issuing authority, and add the following statement:

HR EXCELLENCE IN RESEARCH

UNIWERSYTET PRZYRODNICZY WE WROCŁAWIU
INSTYTUT BIOLOGII ŚRODOWISKOWEJ, ZAKŁAD ANTROPOLOGII
ul. Kożuchowska 5, 51-631 Wrocław
tel. 71 320 58 89 • fax 71 320 58 76
e-mail: antropolgia@upwr.edu.pl • www.antropologia.upwr.edu.pl

'All necessary permits were obtained for the described study, which complied with all relevant regulations.'

If no permits were required, please include the following statement:

'No permits were required for the described study, which complied with all relevant regulations.''

As requested, we've added more precise localization information and specimen numbers to the article: parts 'Site and burial description' (page 5, line 101, 110-115; all page and line numbers refer to the 'Manuscript' file with untracked changes) and 'Individual description' (page 6, line 134-137). I must explain that the subject of our research was obtained during the first excavation season (2019) of the still ongoing project, therefore it has not yet been included in the list of any museum collections records. The numbers derived from the fieldwork documentation attached to the article constitute the best possible formal characteristic of this object at the moment. No permits were required for the described study, which complied with all relevant regulations.

3. *'We note you have included a table to which you do not refer in the text of your manuscript. Please ensure that you refer to Table 1 in your text; if accepted, production will need this reference to link the reader to the Table.'*

We carefully checked all the attached figures and tables and hope that the article meets all the journal's formal standards right now. The Table 1 is mentioned in the 'Cyst analyses' part (page 10, line 251).

4. *'Please review your reference list to ensure that it is complete and correct. If you have cited papers that have been retracted, please include the rationale for doing so in the manuscript text, or remove these references and replace them with relevant current references. Any changes to the reference list should be mentioned in the rebuttal letter that accompanies your revised manuscript. If you need to cite a retracted article, indicate the article's retracted status in the References list and also include a citation and full reference for the retraction notice.'*

As requested, we reviewed the list of references and we are convinced that it is complete and appropriate. Following the suggestions of the Reviewers, we decided to add to the list of references:

- Rühli FJ, Galassi FM, Haeusler M. Palaeopathology: Current challenges and medical impact. Clin Anat. 2016 Oct; 29 (7): 816-22. doi: 10.1002 / ca.22709 (number 6 in Bibliography, mentioned in page 3, line 61)
- Malinowski A, Bożiłow W. Podstawy antropometrii: Metody, techniki, normy. Warszawa-Łódź: Wydawnictwo Naukowe PWN; 1997 (number 47 in Bibliography, mentioned in page 7, line 153).

UNIWERSYTET PRZYRODNICZY WE WROCŁAWIU
INSTYTUT BIOLOGII ŚRODOWISKOWEJ, ZAKŁAD ANTROPOLOGII
ul. Kożuchowska 5, 51-631 Wrocław
tel. 71 320 58 89 • fax 71 320 58 76
e-mail: antropolgia@upwr.edu.pl • www.antropologia.upwr.edu.pl

UNIWERSYTET
PRZYRODNICZY
WE WROCŁAWIU

INSTYTUT BIOLOGII ŚRODOWISKOWEJ
ZAKŁAD ANTROPOLOGII

According to Reviewer #1 comments:

1. *'It is possible to establish the age range or the mean age and the stature for the female individual?'*

Yes, it was possible to establish more precisely the age of the female individual. Based on the morphological details of the skull (cranial suture obliteration) and pelvis (pubic symphysis shape), as well as degenerative changes observed in the bone material, associated with the aging process (presence of articular cartilage degenerative lesions and degeneration of the vertebrae surface), considering that this individual was from a rural population, we estimated age as 45-55 years old. The individual was 158 cm tall, established by Trotter and Gleser method as well as Nainys method. We added this information in the 'Anthropological analyses of the skeleton' section (page 8, line 188-192) of the article.

2. *'I will also add the brand of the machine utilised for X-Rays analyses'*

Of course you are right – thank you very much for pointing out this oversight. The machines used for X-Ray analyses were: a) for SEM: SEM, EVO LS15, Zeiss with energy dispersive X-ray analysis (SEM/EDX) applying quantax detector (Brüker) with 10 kV of filament tension; b) for X-Ray imaging: Konica Minolta Regius Model 110s with Siemens Polydoros LX 30 generator; c) for CT: Siemens Somatom Emotion 16 (3rd generation) apparatus. All the information was added to the 'Methods' part (page 7, line 173-174 & page 8, line 181-184) of the article.

3. *'A 3D virtual reconstruction from the Ct-Scan of the cyst would be very interesting to see (for example a picture with the whole cyst and another one cut in norma sagittalis, so one can appreciate the section)'*

Regarding the inclusion of the three-dimensional reconstruction obtained from CT slices with the article: we agree that it would be an interesting form of object imaging, however, after detailed discussion, we decided that in our case it would not enrich the content of the article. 3D reconstructions are generally performed to reveal a closed object, the structure of which we do not want to break. In this case, we did a CT to view the internal structure of an object that we did not know what it was. After confirming that there were structures resembling human (fetal) bones inside, we extracted them from the inside of the shell to perform the necessary EDS and histological analyzes (which would not have been possible without physical interference with the object). Based on the scans (both in 512 dpi and 1080 dpi resolution), we made a 3D reconstruction, but its quality is not satisfactory (due to the delicate structure of the cyst, the edges of the three-dimensional object were blurred). We believe that this reconstruction does not bring significant informational or aesthetic value to the article, so we preferred to include in the article other images showing the way of our thinking from the general (outer view of burial no. 1095) to the detail (histological stainings and SEM). We hope that you will accept our explanation of this issue and lack of the 3D model does not decrease the value of this article.

UNIWERSYTET PRZYRODNICZY WE WROCŁAWIU
INSTYTUT BIOLOGII ŚRODOWISKOWEJ, ZAKŁAD ANTROPOLOGII
ul. Kożuchowska 5, 51-631 Wrocław
tel. 71 320 58 89 • fax 71 320 58 76
e-mail: antropolgia@upwr.edu.pl • www.antropologia.upwr.edu.pl

UNIWERSYTET
PRZYRODNICZY
WE WROCŁAWIU

INSTYTUT BIOLOGII ŚRODOWISKOWEJ
ZAKŁAD ANTROPOLOGII

<u>According to Reviewer #2 comments:</u>

1. *'I would recommend adding "calcified" to cyst in the title'*

We added the word '*calcified'* to the article's title as suggested.

2. *'I would recommend pointing more on the diagnosis of lithopedion rather than the other one proposed'*

Regarding the suggestion to emphasize the greater possibility that the discovered object is an example of a lithopedion: we cannot fully agree with this proposal as we believe that the information we have does not allow us to fully draw such a conclusion. Although many features of the object indicate its similarity to the diagnostic criteria of lithopedion, the EDS analysis of the shell indicated that it is not composed of calcium, which is known right now as the basic mechanism of lithopedion formation. We agree that lithopedion is more often described in the literature and has already been identified in the archaeological context, however – referring to the review article suggested by the Reviewer – the frequency of publications on a given phenomenon does not necessarily have to be related to the frequency (and therefore higher probability) of its occurrence. We decided to write this article in such a form that it does not resolve this issue unequivocally, in the hope that it will cause a discussion in the literature on the possibility of fetus-in-fetu occurrence in archaeological remains or on the possibility of lithopedion's shell formation by a mechanism other than calcium deposition. We trust that such an approach will contribute to the deepening of the scientific potential. We have provided a brief overview of this explanation in the 'Conclusions' section (page 14, line 327-329) and we hope that the current form of the article will be acceptable.

3. *'I would recommend adding one more general, theoretical reference in the introduction for the workflow of palaeopathological diagnosis and its limitations: Rühli FJ, Galassi FM, Haeusler M. Palaeopathology: Current challenges and medical impact. Clin Anat. 2016 Oct;29(7):816-22. doi: 10.1002/ca.22709.'*

We read the recommended article and agree that it provides valuable support for the content of our theoretical introduction. We referred to it in the 'Introduction' section (position 6 in the Bibliography).

We would like to express our gratitude once again for the careful analysis of our article. We are convinced that thanks to the suggested changes, we were able to improve the quality of both this article and to write our next works in a better way. We are more than happy to address any further issues that might arise from any re-examination of this article.

Best regards

Bisiecka

HR EXCELLENCE IN RESEARCH

UNIWERSYTET PRZYRODNICZY WE WROCŁAWIU
INSTYTUT BIOLOGII ŚRODOWISKOWEJ, ZAKŁAD ANTROPOLOGII
ul. Kożuchowska 5, 51-631 Wrocław
tel. 71 320 58 89 • fax 71 320 58 76
e-mail: antropolgia@upwr.edu.pl • www.antropologia.upwr.edu.pl
